# The next step towards usable microbial bioelectrochemical sensors?

**DOI:** 10.1111/1751-7915.12590

**Published:** 2017-01-06

**Authors:** Jan B. A. Arends

**Affiliations:** ^1^ Faculty of Bioscience Engineering Center for Microbial Ecology and Technology (CMET) Ghent University Coupure Links 653 B‐9000 Gent Belgium

Microbial bioelectrochemical systems have been in the full spotlight for over a decade due to the promise of being a platform for sustainable electrical power generation from wastewaters. This promise has not yet been fulfilled, but the research on bioelectrochemical systems (BES) has received a tremendous boost (Schröder, 2011; Arends and Verstraete, 2012). The field has diversified into exploring various configurations of BESs for energy production/storage, bioremediation/waste clean‐up and bioproduction/electrofermentation, as a tool for fundamental research and for biosensor development (Schröder *et al*., 2015). The fact that many bacteria, with *Geobacter* spp. as one of the model organisms, are (putatively) able to directly deposit electrons on an electrode (Koch and Harnisch, 2016) opens the possibility to translate biologically relevant environmental signals directly into an electrical current. This allows the creation of a low current, amperometric microbial bioelectrochemical sensor system, applicable in many environments. However, to come to practical applications of BES‐based sensors, several issues need to be overcome. Amongst others, one can think of signal/response ratio, signal specificity i.e. minimizing interferences, measurement range, need for calibration, signal stability over time, use of pure cultures or mixed communities, mechanical stability, storage and shelf life of the sensor probe.

The work by Estevez‐Canales *et al*. (2017) as described in their recent manuscript makes a step forward in prolonging the shelf life and optimizing storage conditions of pre‐colonized bioanodes. They have developed a method to colonize an anode with *Geobacter sulfurreducens* in a silica gel matrix embedded in a carbon felt electrode. The microorganisms remained alive within the silica matrix in which they were encapsulated, and their concentration did not change for a minimum of 4 days as assayed with fluorescence microscopy. Activity could be maintained over 18 days in a lactate‐fed system as determined by the current response. Acetate pulse experiments indicated a fast response time, in the order of minutes, of the bacteria encapsulated in the silica matrix. Using pre‐colonized electrodes which take ~1 h to produce (Estevez‐Canales *et al*., 2017) and can be stored offers a great advantage over producing (in the order of days) and possibly storing electroactive biofilms.

As the authors indicate, several issues remain to be addressed to develop storable microbial electrodes for fast, selective and reproducible recordings. For example, optimal storage conditions for these electrodes need to be determined. From the manuscript, it becomes clear that the electrodes were stored under favourable conditions for *Geobacter sulfurreducens*. However, the most extreme conditions for storing the encapsulated microorganisms, in terms of temperature, humidity, time without electron donor/acceptor and exposure to atmospheric oxygen (Lin *et al*., 2004) still need to be understood. It would be interesting to compare the durability of a pre‐colonized electrode with an electroactive biofilm of the same dimensions with regard to their storage conditions but also regarding the mechanical strength of biocatalyst attachment.

An intriguing follow‐up question is the impact of invasion, or colonization, of the silica surface by other microorganisms on the performance of the electrodes with immobilized electroactive microorganisms. Generally, microbial bioelectrochemical sensors are envisioned to be used in ‘dirty’ environments such as (waste)waters, fermenters and anaerobic digesters (Yang *et al*., 2015). These environments are characterized by a very diverse microbial community of which several members might possibly be capable of colonizing the silica matrix and thus hinder analyte mass transfer towards the embedded sensing microorganisms.

On a more positive note, interaction of environmental microorganisms with encapsulated microorganisms (not limited to a *Geobacter* spp. but can be any other electroactive microorganism) on electrodes can allow for specific detection of certain metabolic functions within a sample or environment. Even more, modification of the encapsulation matrix might allow to select for certain molecules to not reach the encapsulated microorganisms, thus creating a protective barrier to (groups of) toxic components.

An important aspect that remains to be addressed is the lower electrochemical activity per cell in the silica matrix. From the study by Estevez‐Canales *et al*. (2017), it becomes clear that the immobilized cells had an averaged specific activity (~10^−1^ fA/cell), which is some orders of magnitude lower compared with other studies (Table 1). This lower specific activity might well be due to the fact that only a fraction of the introduced cells is ‘electrically’ connected to the electrode, that is, not all the encapsulated cells contributed to the detected activity. The authors propose that incorporating conductive material within the encapsulation matrix might increase the overall specific activity of the immobilized cells. A low specific activity is not a problem if comparative (bio)electrochemistry is the aim of using the pre‐colonized electrodes; however, a high specific activity (i.e. current density) is needed when the electrodes are applied for energy production or for highly sensitive biosensors.

**Table 1:** Specific current densities per cell attached to an anode

Current per cell (fA)	Organism	Note	Reference
0.3	*Geobacter sulfurreducens* PCA (DSM 12127)	Re‐calculated from acetate consumption rate of encapsulated cells	Estevez‐Canales *et al*. (2016)
3.1	”	Re‐calculated from acetate consumption rate of attached cells	Estevez‐Canales *et al*. (2015)
92 + /‐ 33	*Geobacter sulfurreducens* DL‐1^a^	Direct measurement on individual cells	Jiang *et al*. (2013)
280	/	Current based on theoretical estimation	Arends and Verstraete (2012)

**a.** Strain DL‐1 is obtained from a single colony of PCA grown on a plate (Coppi *et al*., 2001).

Despite the many challenges that still need to be tackled to come to a usable microbial bioelectrochemical sensor, the work by Estevez‐Canales *et al*. (2017) highlights the possibility of making pre‐colonized, storable electrodes. With encapsulated microbial catalysts, these bioelectrodes can be used for applications/research questions that need reproducibly coated microbial bioelectrodes or fast responses in bioelectrochemical systems.

## Conflict of interest

None declared.
